# Single-port robot-assisted hepatectomy of segments S4b and S5v: A case report

**DOI:** 10.1016/j.iliver.2026.100223

**Published:** 2026-02-04

**Authors:** Xin Lan, Jian Feng, Chenglin Piao, Qiang Li, Zhenduo Si, Chao Zhang, Jianjun Leng

**Affiliations:** Hepatopancreatobiliary Center, Peking University Shougang Hospital, Beijing 100043, China

**Keywords:** Hepatectomy, Single-port robotic, New technology

## Abstract

We report a 63-year-old man with hepatocellular carcinoma who initially underwent transcatheter arterial chemoembolization and two cycles of targeted therapy combined with immunotherapy, which achieved a partial response. He then successfully underwent single-port robot-assisted resection of liver segments S4b and S5v using the SHURUI single-port robotic system. The surgical incision measured 4 cm, intraoperative blood loss was 200 mL, and the operation time was 240 minutes. He recovered well and was discharged on postoperative day 7.

## 1 Introduction

Liver resection is advancing toward minimally invasive and precision surgery, with robotic hepatectomy (RH) is widely accepted. We report a case of hepatectomy assisted by the single-port robotic system (SPRS), discuss its clinical value, and offer insights for clinical application.

## 2 Case presentation

A 63-year-old man was admitted for evaluation of a liver mass found on physical examination, which was confirmed on abdominal ultrasonography. He had no symptoms such as abdominal pain, bloating, fever, or jaundice. His medical history was notable for diabetes, which was well controlled on empagliflozin. Laboratory findings were as follows: HBsAg, HBeAb, and HBcAb were positive; HBV DNA was 1.44 ​× ​10^2^ IU/mL; and AFP was 2.62 ng/mL. His liver function was classified as Child–Pugh class A.

**Fig. 1:**
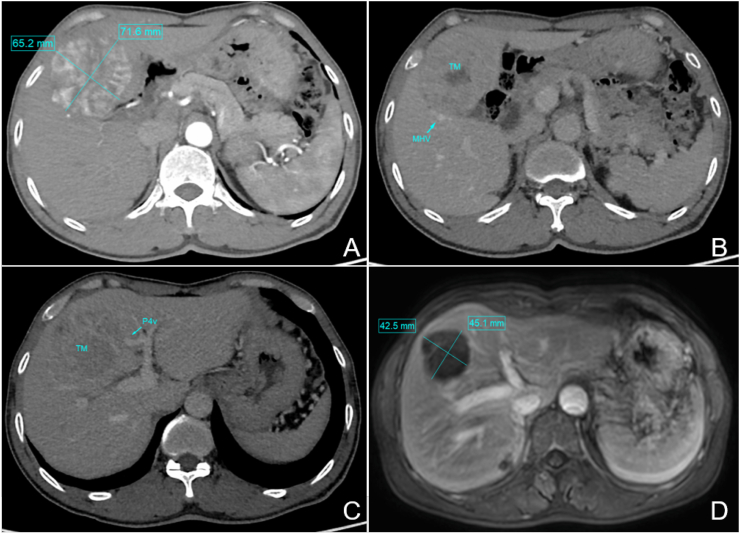
Imaging findings before and after treatment. (A) Before treatment. (B) The tumor was adjacent to the middle hepatic vein (MHV). (C) The tumor was also adjacent to the hepatic pedicle of segment 4 (S4). (D) After neoadjuvant treatment, the tumor shrank considerably.

Contrast-enhanced computed tomography of the abdomen showed a 71 ​× ​65 mm soft tissue density mass in liver segments S4 and S5 that exhibited enhancement in the arterial phase, which decreased in the venous phase (Fig. 1).

**Fig. 2:**
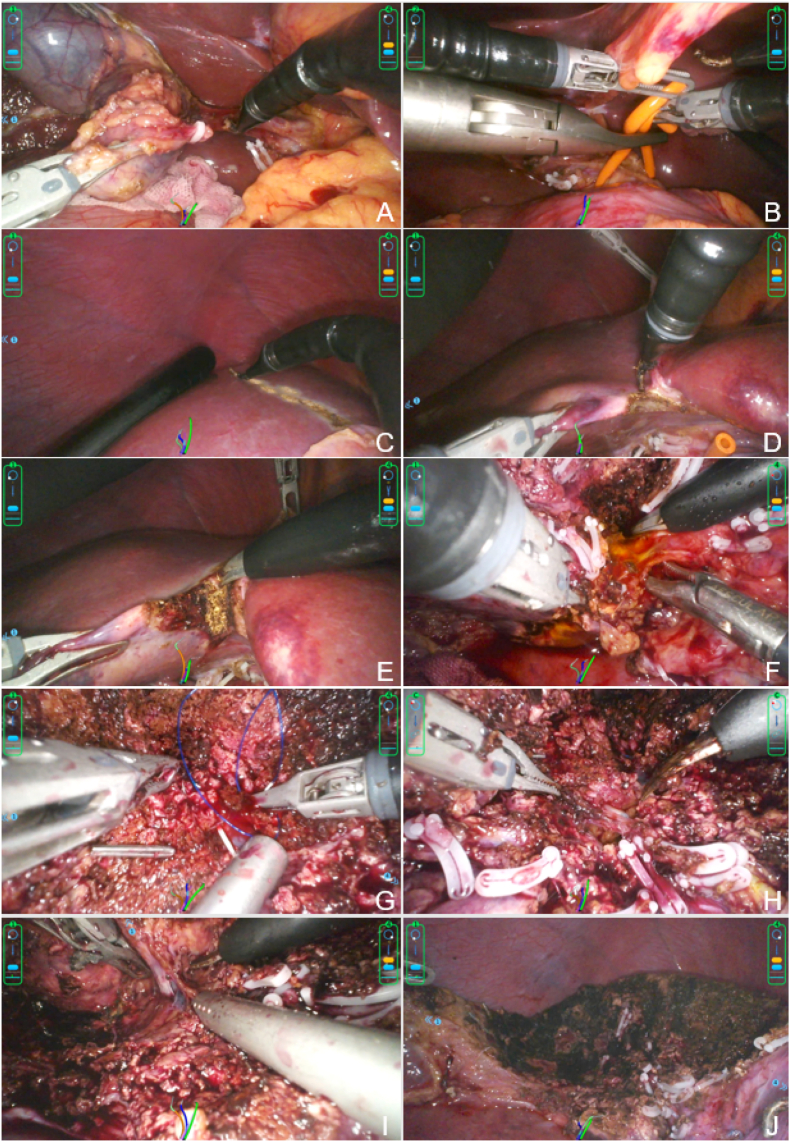
Surgical procedures. (A) Cholecystectomy. (B) Preset hepatic hilum occlusion. (C) Ultrasonic exploration and marking of the hepatectomy line. (D, E) Transection of hepatic parenchyma with electric hook and electric scissors. (F) Ligation of segment 4 posterior (S4p). (G) Suture ligation of the umbilical fissure vein (UFV). (H) Ligation of segment 5 posterior vein (S5Vp). (I) Ligation of middle hepatic vein segment 4 (MHV4). (J) Postoperative surgical wound.

**Fig. 3:**
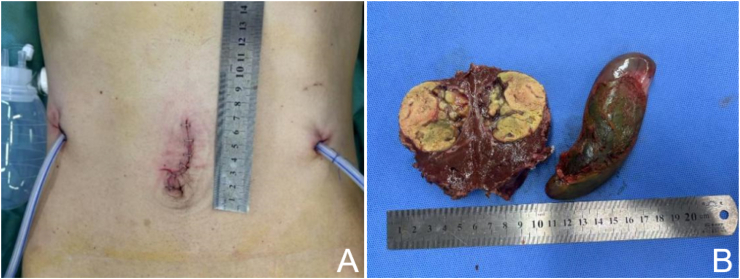
(A) A 4 cm surgical incision. (B) Surgical specimen.

After initial treatment with transcatheter arterial chemoembolization and intravenous sintilimab/bevacizumab, follow-up liver magnetic resonance imaging showed tumor regression, consistent with a partial response. We then performed single-port robot-assisted resection of liver segments S4b ​+ ​S5v (Fig. 2). Intraoperative blood loss was 200 mL. Operation time was 240 minutes. No postoperative complications occurred. The patient gradually resumed oral intake. He was allowed to ambulate the day after surgery. The drainage tube was removed on postoperative day 3. He was discharged on postoperative day 7 (see Fig. 3).

Histopathologically, 95% of the tumor was necrotic; the remainder was moderately differentiated, with no microvascular invasion. The tumor capsule was intact. The resection was judged to be complete (R0). Cirrhosis was also apparent. The patient was recurrence-free at the 6-month follow-up.

The Peking University Shougang Hospital Ethics Committee approved this study, with ethical approval number IRBK-2025-093-01. Written informed consent for the publication of this case report has been obtained from the patient himself.

## 3 Literature review and discussion

In 2003, Giulianotti et al. first reported robotic hepatectomy.^1^ Subsequent studies have confirmed the feasibility and safety of robotic surgical systems in liver surgery. The release of the 2023 International Expert Consensus on Robotic Hepatectomy indicates that RH has been widely accepted and standardized.^2^ The SPRS combines the precision of robotic surgical systems with the minimally invasive advantages of single-port surgery, is being applied in urology, gastrointestinal surgery, and thoracic surgery.^3^, ^4^, ^5^ To the best of our knowledge, we are the first to report complete resection of liver segments S4b ​+ ​S5v using the SHURUI (Beijing Surgerii Robotics Company Limited, Beijing, China) SPRS (Sci-Tech Novelty Retrieval Report No.: 202536000Z140184).

The SHURUI SPRS offers multiple advantages in clinical practice. SPRS features an ultra-minimally invasive incision, shortens operation duration, and accelerates postoperative recovery,^6^, ^7^, ^8^ showing potential to replace multi-port robots and single-port laparoscopes for certain surgical procedures. In our patient, a small 4-cm periumbilical midline abdominal incision was used. This incision also served as a channel for specimen extraction, and a drainage tube was placed through the auxiliary port—no additional incisions were needed compared with the traditional five-port method. In comparison with previously published data on multi-port robotic hepatectomy and laparoscopic hepatectomy cases from our institution, this case exhibited similar operation time and intraoperative blood loss.^9^^,^^10^

SPRS is particularly suitable for precise dissection in narrow spaces. During the operation, we sequentially dissected and ligated the S4 hepatic pedicle, umbilical fissure vein, S5v, and the S4 segment branch of the middle hepatic vein. Only one vascular suture was performed throughout the operation, and the experience and fluency of the suture operation were significantly better than those of laparoscopy. The reported average docking time of the da Vinci surgical system is 7 to 10 minutes, while the docking time in this study was only 5 minutes.^11^^,^^12^ In addition, the one-click lens cleaning function, an innovation of the SHURUI SPRS, can also save operation time and improve efficiency. Based on the 20 cases completed by the operating surgeon to date, surgeons with extensive experience in multi-port robot-assisted hepatectomy can rapidly master the operation techniques of SPRS.

Compared with traditional laparoscopy, the SPRS surgical arm can achieve 120° all-round free movement, highly simulating the flexibility of human hands and avoiding the “chopstick effect” of single-port laparoscopic instruments. This advantage is particularly prominent during preset hepatic hilum blockade—the bendable surgical arm can attempt to pass through the posterior space of the hepatic hilum from multiple angles. However, because of the lack of force feedback, gentle operation should be emphasized to avoid iatrogenic injury.

The SHURUI SPRS has some limitations. SPRS completely avoids the collision risk of external surgical arms and greatly reduces the requirements for port placement. However, the operating space of the auxiliary port is relatively narrow. The authors suggest that when performing SPRS hepatectomy, the auxiliary port can be placed 1 cm below the costal margin on the right midaxillary line. On the other hand, laparoscopic and multi-port robotic hepatectomies mainly use ultrasonic scalpels as the primary energy device. However, because of limitations in channel capacity and energy transmission, SPRS is not yet equipped with ultrasonic scalpels. Although the liver parenchyma was successfully transected in this case using an electric scissor combined with bipolar electrocoagulation, the substantial advantages of ultrasonic scalpels in liver parenchyma transection (e.g., clamping, dissection, and especially controlling minor vascular bleeding) cannot be denied. We suggest that, when necessary, an ultrasonic scalpel can be used through the auxiliary port to assist in liver parenchyma transection.

Based on our accumulated case experience, the ideal indications for SPRS hepatectomy are as follows: tumors ≤5 cm in diameter located in the S2, S3, S4, S5 or S6 segments; and no extensive vascular invasion. This approach is particularly recommended for patients with a strong preference for optimal cosmetic results.

## 4 Summary

The SHURUI SPRS has achieved some technological breakthroughs and improved the accessibility of clinical application of single-port robots. With the improvement of instruments, as well as the accumulation of experience by surgeons, the application prospects of SPRS in hepatectomy are expected to broaden.

## CRediT authorship contribution statement

**Xin Lan:** Writing – original draft, Methodology, Data curation. **Jian Feng:** Writing – original draft, Data curation. **Chenglin Piao:** Methodology, Data curation. **Qiang Li:** Methodology, Data curation. **Zhenduo Si:** Data curation, Visualization. **Chao Zhang:** Visualization, Validation. **Jianjun Leng:** Writing – review & editing, Conceptualization, Supervision.

## Informed consent

Written informed consent for the publication of this case report has been obtained from the patient himself.

## Data availability statement

Patient imaging and clinical data are stored in the electronic medical records system of Peking University Shougang Hospital and can be requested from the corresponding author, subject to hospital approval.

## Ethics statement

The Peking University Shougang Hospital Ethics Committee approved this study, with ethical approval number IRBK-2025-093-01.

## Declaration of generative AI and AI-assisted technologies in the writing process

No AI-tools were utilized at any stage of drafting and revising this manuscript.

## Funding

None

## Declaration of competing interest

The authors declare no competing interests.

## Abbreviations

SPRS: single-port robotic system
RH: robotic hepatectomy
